# Insulin Deintensification After Glucagon-Like Peptide-1 (GLP-1)-Based Therapy in Type 2 Diabetes: Evidence and a Practical Clinical Framework

**DOI:** 10.7759/cureus.117614

**Published:** 2026-10-07

**Authors:** Ahmed Elhassan, Abu-Bekr Mohamed, Ahmed Mahgoub, Anaida Singh, Isra Ali

**Affiliations:** 1 Endocrinology, Mohammed Bin Rashid University of Medicine and Health Sciences, Dubai, ARE; 2 Internal Medicine, Detroit Medical Center/Wayne State University, Detroit, USA; 3 Anatomy, University of Khartoum, Khartoum, SDN; 4 Medical School, St. George's University, St. George's, GRD; 5 Biomedical Sciences, Mohammed Bin Rashid University of Medicine and Health Sciences, Dubai, ARE

**Keywords:** glp-1 receptor agonist, insulin deintensification, insulin discontinuation, treatment simplification, type 2 diabetes

## Abstract

Glucagon-like peptide-1 (GLP-1)-based therapies have changed the management of insulin-treated type 2 diabetes by creating opportunities not only to improve glycemic control and body weight but also to reduce insulin exposure and simplify previously intensified treatment regimens. However, clinical guidance remains considerably clearer on when to initiate or intensify insulin than on how to safely reduce established insulin therapy after effective GLP-1-based treatment is introduced.

This narrative clinical review examines evidence for insulin dose reduction, regimen simplification, prandial insulin withdrawal, and complete insulin withdrawal following initiation or intensification of GLP-1-based therapy in adults with type 2 diabetes. Randomized trials and real-world studies increasingly support simplification of complex insulin regimens, with the strongest direct evidence supporting reduction or withdrawal of prandial insulin in appropriately selected patients. Complete insulin withdrawal is achievable in some individuals but remains less extensively studied.

A practical approach should distinguish basal from prandial insulin and integrate overall glycemic control with fasting and postprandial glucose patterns, hypoglycemia risk, current insulin requirements, and clinical features suggesting impaired endogenous insulin reserve. Continuous glucose monitoring or structured glucose monitoring can facilitate sequential adjustment as GLP-1-based therapy is titrated. Patients with marked hyperglycemia, previous ketosis or diabetic ketoacidosis, suspected autoimmune diabetes, or other evidence of clinically important insulin deficiency require greater caution.

We propose an evidence-informed phenotype-based framework in which insulin components are progressively reassessed rather than routinely continued or withdrawn according to a fixed formula. The objective is not insulin independence itself, but identification of the least complex insulin regimen necessary to maintain safe, individualized glycemic control.

## Introduction and background

Insulin remains an essential and highly effective treatment for type 2 diabetes when non-insulin therapies are insufficient to achieve individualized glycemic goals. Traditionally, insulin treatment has followed a predominantly intensification-oriented pathway: basal insulin is initiated first, followed when necessary by one or more prandial injections and ultimately a basal-bolus or multiple-daily-injection (MDI) regimen. Although effective, increasing insulin intensity may increase injection and monitoring burden, hypoglycemia risk, treatment complexity, and weight gain.

Basal insulin primarily controls fasting and between-meal glucose by suppressing hepatic glucose production, whereas prandial insulin is used to limit meal-related glucose excursions. This distinction is important when considering deintensification because improvement after GLP-1-based therapy may reduce the need for these insulin components to different degrees.

The increasing efficacy and use of glucagon-like peptide-1 receptor agonists (GLP-1 RAs) and dual glucose-dependent insulinotropic polypeptide (GIP)/GLP-1 RAs have altered this treatment landscape. These therapies can substantially improve glycemic control while promoting weight loss and carrying relatively low intrinsic hypoglycemia risk. Through glucose-dependent enhancement of insulin secretion, suppression of inappropriate glucagon secretion, effects on gastric emptying and appetite, and associated weight loss, these therapies can reduce postprandial glucose excursions and overall exogenous insulin requirements. Contemporary diabetes management therefore increasingly requires reassessment of existing insulin requirements when potent GLP-1-based therapy is introduced [1]. The term GLP-1-based therapy is used pragmatically here to encompass GLP-1 RAs and dual GIP/GLP-1 RAs when discussing shared implications for insulin requirements; findings from individual agents are attributed to the therapy studied rather than assumed to represent a class-wide effect.

Early randomized trials demonstrated that GLP-1 RA-based treatment could provide an alternative to adding prandial insulin to basal therapy [2-6]. Subsequent direct deintensification trials and observational studies have shown that established basal-bolus or MDI regimens can be simplified and that prandial insulin can be reduced or withdrawn in appropriately selected patients [7-13]. These findings raise an important clinical question: once effective GLP-1-based therapy changes a patient's glycemic physiology and insulin requirement, how should a previously intensified insulin regimen be reassessed?

This review therefore aims to distinguish the different forms of insulin deintensification, critically appraise the evidence supporting each, identify clinical features that may influence feasibility and safety, and translate the available evidence into a practical framework for reassessing established insulin therapy after GLP-1-based treatment is introduced.

Scope and terminology

In this review, insulin deintensification is used as an umbrella term encompassing reduction in insulin dose or treatment complexity after initiation or intensification of GLP-1-based therapy. Prandial insulin withdrawal refers specifically to discontinuation of mealtime insulin while basal insulin is retained, whereas complete insulin withdrawal refers to discontinuation of all exogenous insulin. Trials in which GLP-1-based therapy was used instead of adding prandial insulin are classified as avoidance of insulin intensification rather than direct deintensification. These outcomes are analyzed separately because they represent clinically and evidentially distinct treatment strategies.

Literature review approach

This narrative clinical review was developed through targeted PubMed/MEDLINE searches and backward citation searching of relevant primary studies, clinical guidance, and focused reviews, with the literature updated through August 2026. Search concepts included combinations of “type 2 diabetes,” “GLP-1 receptor agonist,” “semaglutide,” “tirzepatide,” “fixed-ratio combination,” “insulin,” “basal-bolus,” “multiple daily injections,” “deintensification,” “simplification,” “prandial insulin withdrawal,” and “insulin discontinuation.” Priority was given to randomized controlled trials directly evaluating simplification or withdrawal of established insulin therapy, followed by observational studies addressing durability and potential determinants of successful deintensification. Randomized trials comparing GLP-1-based therapy with initiation of prandial insulin were considered separately as evidence for avoidance of insulin intensification. Contemporary clinical guidance and focused deprescribing literature were used to inform insulin-adjustment and monitoring principles. The narrative design was selected to integrate heterogeneous randomized, observational, guideline, and practical evidence into a clinically oriented framework rather than estimate a pooled treatment effect. This review did not employ a systematic-review protocol, formal risk-of-bias assessment, or meta-analysis. Formal certainty-of-evidence ratings were not assigned. Evidence was interpreted according to study design, directness to the deintensification question, population selection, sample size, follow-up duration, and major methodological limitations. Randomized trials were given greater weight for causal assessment of specific deintensification strategies, whereas observational cohorts were used primarily to inform real-world feasibility, durability, and potential predictors of successful deintensification. This qualitative hierarchy should not be interpreted as a formal Grading of Recommendations, Assessment, Development and Evaluations (GRADE) assessment.

## Review

From avoiding intensification to reversing it

The earliest relevant randomized evidence demonstrated that GLP-1 RA therapy could substitute for progression toward prandial insulin in patients inadequately controlled with basal insulin. The 4B, Harmony 6, and GetGoal Duo-2 trials established that GLP-1 RA-based strategies could provide glycemic efficacy comparable to conventional prandial insulin intensification while generally favoring weight and hypoglycemia outcomes [2-4]. DUAL VII demonstrated that a fixed-ratio basal insulin/GLP-1 RA combination could achieve glycemic control comparable to basal-bolus treatment with substantially lower insulin exposure [5]. SURPASS-6 extended this concept using tirzepatide, demonstrating superior glycemic and weight outcomes compared with the addition of prandial insulin lispro to basal insulin [6]. The greater glycemic and weight effects observed with dual GIP/GLP-1 receptor agonism raise the possibility of a greater insulin-sparing effect than with some selective GLP-1 RAs. However, SURPASS-6 primarily demonstrated avoidance of prandial insulin intensification rather than withdrawal of already established prandial insulin. Direct comparative evidence showing that tirzepatide permits greater or faster withdrawal of established insulin than selective GLP-1 RAs remains insufficient. Deintensification findings should therefore remain agent- and study-specific rather than being extrapolated solely from relative glucose- or weight-lowering efficacy [6]. These studies establish that escalation to complex prandial insulin treatment can frequently be avoided, but they should not be interpreted as direct evidence that established prandial insulin can be withdrawn.

Direct evidence for insulin deintensification

The BEYOND pragmatic randomized trial demonstrated that established basal-bolus therapy could be simplified to less complex treatment strategies without sacrificing glycemic improvement [7]. Rosenstock et al. evaluated planned withdrawal of established prandial insulin after introduction of weekly albiglutide; prandial insulin was initially reduced by 50% and subsequently discontinued, providing direct randomized evidence for a staged withdrawal strategy [8]. TRANSITION-T2D enrolled 60 adults with glycated hemoglobin (HbA1c) ≤7.5% receiving MDI with total daily insulin ≤120 units/day. At 26 weeks, 97.5% of participants in the semaglutide arm had stopped prandial insulin and total daily insulin had fallen by 56%, with similar hypoglycemia frequency between groups [9]. The study is compelling proof of concept but was small, single-center, open-label, and deliberately selected; its results should not be extrapolated indiscriminately.

IDEAL: Broadening randomized deintensification evidence

The 2026 IDEAL trial evaluated transition from MDI to once-daily iGlarLixi (insulin glargine/lixisenatide). Eligible participants had HbA1c ≤9%, total daily insulin ≤0.8 IU/kg, and fasting C-peptide above the lower limit of normal. Ninety participants were analyzed over 24 weeks. Glycemic control was comparable between groups, while iGlarLixi produced between-group advantages of 28.57 IU/day in total insulin dose and 4.19 kg in body weight [10]. IDEAL broadens randomized evidence beyond the near-target population studied in TRANSITION-T2D and supports fixed-ratio basal insulin/GLP-1 RA therapy as a direct simplification strategy in selected patients.

The principal randomized and observational evidence informing this hierarchy is summarized in Table 1.

**Table 1:** Evidence informing insulin deintensification after GLP-1-based therapy in adults with type 2 diabetes Avoidance of intensification, direct simplification, prandial withdrawal, and complete insulin withdrawal are distinct outcomes. Abbreviations: BBI, basal-bolus insulin; MDI, multiple daily injections; RCT, randomized controlled trial; GLP-1 RA, glucagon-like peptide-1 receptor agonist; HbA1c, glycated hemoglobin; wk, weeks; mo, months; iDegLira, insulin degludec/liraglutide; iGlarLixi, insulin glargine/lixisenatide​​​​​​*.*

Study	Population/design	Strategy	Follow-up	Principal insulin outcome	Evidence role
4B [2]	Basal insulin; RCT	Exenatide vs adding lispro	30 wk	Avoided bolus intensification	Avoidance of intensification
Harmony 6 [3]	Basal insulin; RCT	Albiglutide vs adding lispro	26 wk	Alternative to prandial intensification	Avoidance of intensification
GetGoal Duo-2 [4]	Basal insulin; RCT	Lixisenatide vs glulisine	26 wk	Alternative to insulin escalation	Avoidance of intensification
DUAL VII [5]	Basal insulin; RCT	IDegLira vs basal-bolus	26 wk	Comparable HbA1c with lower insulin exposure	Alternative/simplification
SURPASS-6 [6]	Basal insulin; RCT	Tirzepatide vs adding lispro	52 wk	Avoided prandial intensification	Avoidance of intensification
BEYOND [7]	Established BBI; pragmatic RCT	Simplification from BBI	6 mo	Simpler regimen with preserved glycemic improvement	Direct simplification
Rosenstock 2020 [8]	Basal-prandial; RCT	50% bolus reduction then withdrawal	26 wk	54% replaced prandial insulin	Direct staged withdrawal
TRANSITION-T2D [9]	Well-controlled MDI; RCT	Semaglutide + basal; immediate bolus withdrawal	26 wk	97.5% stopped prandial insulin	Direct immediate withdrawal
IDEAL [10]	Established MDI; RCT	MDI to once-daily iGlarLixi	24 wk	Comparable HbA1c; lower insulin dose	Direct randomized simplification
Bonora 2021 [11]	BBI; multicenter routine care	GLP-1 RA deintensification	~14 mo	58.6% stopped prandial insulin	Observational feasibility
Falcetta 2023 [12]	BBI; observational	Long-term deintensification	24 mo	81% stopped prandial; 38.6% also stopped basal	Long-term observational
GLIMPLES 2025 [13]	674 insulin-treated adults	Once-weekly semaglutide	Median 18 mo	32.8% stopped all insulin; 75% of BBI users stopped bolus	Contemporary real-world evidence

Evidence hierarchy

Randomized evidence is strong for avoiding progression to prandial insulin and increasingly strong for simplifying established intensive insulin regimens. Direct randomized evidence supports withdrawal of established prandial insulin in selected populations, whereas longer-term observational studies provide evidence regarding durability and further basal reduction. Evidence supporting complete withdrawal of all insulin remains less extensive. These endpoints should not be considered interchangeable.

Study design alone does not eliminate important limitations. Several randomized deintensification trials were relatively small, open-label, short in duration, or conducted in deliberately selected populations, whereas larger observational cohorts offer broader real-world representation but remain susceptible to confounding and treatment-selection bias. The two forms of evidence are therefore complementary: randomized trials better establish whether a strategy can produce a treatment effect under defined conditions, while observational studies better characterize feasibility and durability in routine clinical populations.

Long-term and real-world evidence

In routine care, Bonora et al. reported that 58.6% of 425 patients deintensified basal-bolus insulin after GLP-1 RA initiation [11]. Falcetta et al. followed 156 participants for 24 months; 81% discontinued prandial insulin and 38.6% also discontinued basal insulin, with baseline insulin requirement emerging as the significant predictor of prandial discontinuation [12]. The 2025 GLIMPLES study included 674 insulin-treated adults. During a median 18-month follow-up, 32.8% discontinued insulin completely, while 75% of those initially receiving basal-bolus insulin discontinued bolus insulin [13].

Which insulin component should be reassessed first?

Basal and prandial insulin serve different physiological functions and should not be treated as a single total daily insulin exposure. In patients receiving basal-bolus therapy, the strongest direct evidence supports preferential reassessment of prandial insulin [7-10]. Successful withdrawal of prandial insulin does not imply that basal insulin is unnecessary. A coherent sequence is therefore basal-bolus therapy → reduce or withdraw unnecessary prandial insulin → establish stability on basal insulin plus GLP-1-based therapy → reassess basal requirement longitudinally → consider complete withdrawal only when clinically appropriate.

Who is most likely to deintensify successfully?

No validated prediction model currently determines whether an individual patient can discontinue prandial or all insulin. Lower baseline insulin requirements represent one of the more consistent signals in observational evidence [11-13]. Several cohort analyses associate shorter diabetes duration and better baseline glycemic control with successful insulin reduction [11,13]. However, available observational evidence does not establish diabetes duration as an independent causal predictor across populations. Shorter duration may partly act as a surrogate for greater residual beta-cell function, lower insulin requirements, or less advanced disease. Diabetes duration should therefore be regarded as a probability modifier rather than an independent eligibility criterion or threshold. A small retrospective study found that participants with normal random C-peptide were more likely to discontinue insulin, but the sample was limited and its numerical threshold should not be converted into a universal cutoff [14]. IDEAL also incorporated preserved fasting C-peptide into eligibility [10].

C-peptide testing is not required before every insulin dose reduction or withdrawal of an individual prandial dose. However, when complete insulin discontinuation is being considered, C-peptide should be measured to exclude severe insulin deficiency, particularly when diabetes classification or endogenous insulin reserve is uncertain [15]. C-peptide should be interpreted together with the concurrent glucose concentration and clinical context rather than as an isolated numerical cutoff. A low C-peptide should prompt caution regarding major insulin withdrawal, whereas a preserved value may support but does not by itself establish that insulin can be safely discontinued.

Screening for possible autoimmune diabetes before major insulin withdrawal

Routine islet-autoantibody testing is not necessary before every attempt to reduce prandial insulin in a patient with established type 2 diabetes. However, diabetes classification should be reconsidered before major insulin withdrawal when clinical features raise concern for autoimmune diabetes or clinically important insulin deficiency. These include younger age at diagnosis, relatively rapid progression to insulin dependence, previous ketosis or diabetic ketoacidosis, unexplained weight loss or other catabolic features, and a clinical course atypical for type 2 diabetes [15]. When autoimmune diabetes is suspected, glutamic acid decarboxylase (GAD) should be the primary antibody measured; if negative, islet antigen 2 (IA-2) and/or zinc transporter 8 (ZnT8) antibodies can be considered when available [15]. C-peptide provides complementary information regarding endogenous insulin secretion. Major insulin withdrawal should be deferred when clinically important insulin deficiency remains possible.

Clinical characteristics that may influence feasibility and safety are summarized in Table 2.

**Table 2:** Clinical factors potentially influencing the feasibility and safety of insulin deintensification These characteristics are probability and safety modifiers rather than validated eligibility criteria. Abbreviations: LADA, latent autoimmune diabetes in adults; CGM, continuous glucose monitoring; SMBG, self-monitoring of blood glucose; DKA, diabetic ketoacidosis; HbA1c, glycated hemoglobin.

Clinical factor	Evidence interpretation	Practical implication
Lower baseline insulin requirement [11-13]	Most consistent favorable observational signal	Greater probability of substantial deintensification
Shorter diabetes duration [11,13]	Associated with success in observational cohorts	Probability modifier; may partly reflect preserved beta-cell reserve
Lower baseline HbA1c [11,13]	Associated with success in observational cohorts	Probability modifier, not threshold
Preserved endogenous insulin secretion	Biologically plausible; limited evidence	Consider C-peptide particularly before complete withdrawal or when insulin deficiency is uncertain; no universal deintensification cutoff [15]
Fasting/nocturnal hypoglycemia	Suggests excessive basal exposure	Reassess basal insulin
Postprandial hypoglycemia	Suggests excessive meal-related exposure	Reassess prandial insulin
Marked persistent hyperglycemia	Safety concern	Preserve adequate insulin initially
Previous DKA/recurrent ketosis	Major safety concern	Avoid aggressive withdrawal until phenotype clarified
Suspected autoimmune diabetes/LADA	Possible insulin deficiency	Clarify phenotype before major withdrawal [15]
Reliable CGM/structured SMBG	Facilitates dynamic adjustment	Improves monitoring rather than predicting success

Why GLP-1-based therapy creates an opportunity for deintensification

The rationale for insulin deintensification is not simply that glucagon-like peptide-1 receptor agonists lower glycated hemoglobin. Their glucose-dependent enhancement of insulin secretion, suppression of inappropriate glucagon secretion, effects on gastric emptying, reduction in appetite, and associated weight loss can reduce postprandial glucose excursions and overall exogenous insulin requirements. These effects are particularly relevant when prandial insulin was added during an earlier phase of poorer metabolic control and subsequently becomes disproportionate to current physiology. The low intrinsic hypoglycemia risk of GLP-1-based therapy also shifts the risk-benefit balance away from maintaining insulin doses that no longer provide meaningful glycemic benefit [1,16].

This does not mean that GLP-1-based therapy substitutes for insulin in every patient. Basal insulin primarily restrains fasting and between-meal hepatic glucose output, whereas prandial insulin addresses meal-related excursions. A patient may therefore lose the need for mealtime insulin while continuing to require basal insulin. This physiological distinction helps explain why prandial withdrawal has emerged as the most reproducible form of direct deintensification in randomized and real-world studies [7-13].

Immediate versus staged prandial insulin withdrawal

Published trials demonstrate more than one workable route to prandial insulin withdrawal. Rosenstock et al. used a staged strategy in which prandial insulin was initially reduced before attempted discontinuation [8]. By contrast, TRANSITION-T2D tested immediate replacement of prandial insulin with weekly semaglutide in a deliberately selected population with relatively well-controlled diabetes and a total daily insulin dose of 120 units/day or less [9]. These approaches should not be viewed as competing universal protocols. Rather, they illustrate that the intensity of the initial reduction can be matched to glycemic stability and the likelihood of residual insulin requirement.

A staged approach is conceptually preferable when baseline glycemia remains above target, insulin requirements are substantial, glucose data are limited, or there is uncertainty regarding endogenous insulin reserve. More decisive prandial withdrawal may be reasonable when glycemia is already near target, meal-related insulin exposure appears excessive, monitoring is reliable, and there are no clinical features suggesting insulin deficiency. The central principle is adaptive rather than formulaic: the safer starting strategy depends on the patient's current glucose and insulin phenotype.

Fixed-ratio combinations as a simplification strategy

Fixed-ratio combinations of basal insulin and a GLP-1 receptor agonist occupy an important middle position between complex basal-bolus therapy and complete insulin withdrawal. DUAL VII showed that insulin degludec/liraglutide could achieve glycemic control comparable to basal-bolus therapy with lower insulin exposure [5]. BEYOND subsequently demonstrated in a pragmatic setting that a complex basal-bolus regimen could be replaced by a once-daily fixed-ratio basal insulin/GLP-1 receptor agonist strategy while maintaining glycemic improvement and reducing treatment complexity [7]. IDEAL further strengthened direct randomized evidence for transition from multiple daily injections to once-daily insulin glargine/lixisenatide in selected patients [10].

These findings are clinically important because simplification need not be equated with insulin elimination. For many patients, the optimal endpoint may be preservation of basal insulin within a simpler GLP-1-based regimen rather than pursuit of complete insulin independence. This distinction provides a more realistic treatment goal for patients with residual fasting insulin requirements.

Fixed-ratio combinations also have an inherent practical limitation because basal insulin and the GLP-1 receptor agonist are delivered in a fixed proportion and therefore cannot be titrated independently. Product-specific dosing ranges may further limit their suitability when substantially different adjustment of the two components is required [17]. These combinations may therefore be particularly useful when the required doses of both components fall within the available titration range, but less suitable when independent titration is clinically important.

Patient-centered outcomes and treatment burden

The value of deintensification extends beyond glycated hemoglobin. Multiple daily injections impose injection burden, glucose-monitoring demands, meal-timing constraints, and fear of hypoglycemia. In BEYOND, simplification reduced daily insulin exposure and injections and was associated with less hypoglycemia than continued basal-bolus intensification [7]. TRANSITION-T2D reported a trend toward greater treatment satisfaction after replacement of prandial insulin with weekly semaglutide [9]. These findings support treatment burden as a legitimate outcome rather than a secondary convenience.

Deintensification should nevertheless remain preference-sensitive. Some patients may prioritize fewer injections, whereas others may prefer the predictability of an established insulin regimen or may experience adverse effects that limit GLP-1-based therapy. The clinically successful regimen is therefore not necessarily the regimen with the fewest injections; it is the least burdensome regimen that remains safe, effective, tolerable, and acceptable to the individual.

Novel clinical synthesis: deintensification as a hierarchy rather than a binary endpoint

A central limitation of the literature is that deintensification has been used to describe several biologically different outcomes. Dose reduction, fewer injections, withdrawal of prandial insulin, reduction of basal insulin, and complete insulin discontinuation should not be collapsed into a single success endpoint. The evidence is strongest for avoidance of prandial intensification and for simplification or withdrawal of prandial insulin in selected patients; it becomes progressively less certain as the target moves toward complete insulin withdrawal [2-13].

We therefore propose a hierarchical model of insulin deintensification: first remove demonstrably excessive insulin exposure; next reduce injection complexity; preferentially reassess prandial insulin when meal-related coverage is no longer required; reassess basal insulin only after fasting and overnight stability is established; and consider complete withdrawal only when glycemic control remains stable without physiologically necessary insulin. This hierarchy reframes deintensification from a one-time decision into repeated physiological reassessment.

No randomized trial has prospectively evaluated this complete sequence as a single linear algorithm. The proposed hierarchy integrates evidence from studies addressing different components of deintensification and should therefore be interpreted as an iterative clinical framework rather than a mandatory stepwise pathway. Depending on the patient's insulin and glycemic phenotype, individual stages may occur concurrently, be repeated, or not be required.

A second implication is that treatment failure should not be defined as inability to discontinue all insulin. A patient who moves from four injections daily to one basal injection while maintaining individualized glycemic control has achieved meaningful deintensification even if basal insulin remains necessary. Conversely, complete withdrawal that leads to recurrent hyperglycemia or ketosis is not a successful outcome. This distinction may improve both clinical decision-making and the design of future trials.

From evidence to clinical application

The literature supports progressively greater degrees of deintensification: dose reduction → fewer injections → prandial withdrawal → basal reduction → selected complete withdrawal. The strength of direct evidence decreases toward complete insulin withdrawal. The clinical objective should therefore be to identify insulin that has become physiologically unnecessary while preserving insulin that remains necessary.

Proposed evidence-informed clinical framework

A practical framework should integrate glycemic phenotype (overall control, fasting/overnight glucose, postprandial excursions, hypoglycemia, and CGM patterns when available); insulin phenotype (basal only, basal-plus, or basal-bolus/MDI); and insulin-deficiency/safety phenotype (previous diabetic ketoacidosis (DKA) or recurrent ketosis, suspected autoimmune diabetes, catabolic symptoms, marked persistent hyperglycemia, or unexpected deterioration after insulin reduction).

Terms such as “near target” and “above target” refer to the individual's agreed glycemic goal rather than a universal HbA1c threshold. This wording is intentional because deintensification trials used heterogeneous eligibility criteria [9,10] and contemporary glycemic targets are individualized [1]. Trial-specific numerical thresholds are reported when describing the corresponding studies but should not be converted into universal eligibility criteria for clinical deintensification.

These considerations are integrated into a practical approach in Table 3 and Figure 1.

**Table 3:** Proposed evidence-informed approach to insulin reassessment after GLP-1-based therapy Authors' evidence-informed synthesis; not a prospectively validated treatment algorithm. Abbreviations: BBI, basal-bolus insulin; MDI, multiple daily injections; DKA, diabetic ketoacidosis; CGM, continuous glucose monitoring; SMBG, self-monitoring of blood glucose; GLP-1, glucagon-like peptide-1.

Clinical phenotype	Initial insulin approach	Monitoring	Subsequent strategy
Basal only + near target	Consider modest basal reduction	Fasting/overnight	Further reduce if insulin excess develops
Basal only + fasting hyperglycemia	Preserve adequate basal coverage	Fasting glucose	Reassess during GLP-1 titration
Basal-plus + controlled glycemia	Preferentially reassess prandial component	Postprandial	Withdraw prandial if stable
BBI/MDI + near target + no major insulin-deficiency features	Major prandial reduction or withdrawal may be considered	CGM/structured SMBG	Maintain basal initially; reassess later
BBI/MDI + above target	Favor staged prandial reduction	Fasting + postprandial	Sequential simplification
Fasting/nocturnal hypoglycemia	Reduce corresponding basal exposure	Overnight/fasting	Reassess before further reduction
Postprandial hypoglycemia	Reduce corresponding prandial insulin	Meal-related	Consider withdrawal if control persists
Marked hyperglycemia	Avoid routine aggressive withdrawal	Overall trajectory	Reassess after metabolic improvement
Suspected insulin deficiency/DKA history	Preserve adequate insulin	Ketosis + glycemia	Clarify phenotype before major reduction
Successful prandial withdrawal	Continue basal + GLP-1-based therapy initially	Fasting/overnight	Reassess basal longitudinally

**Figure 1:**
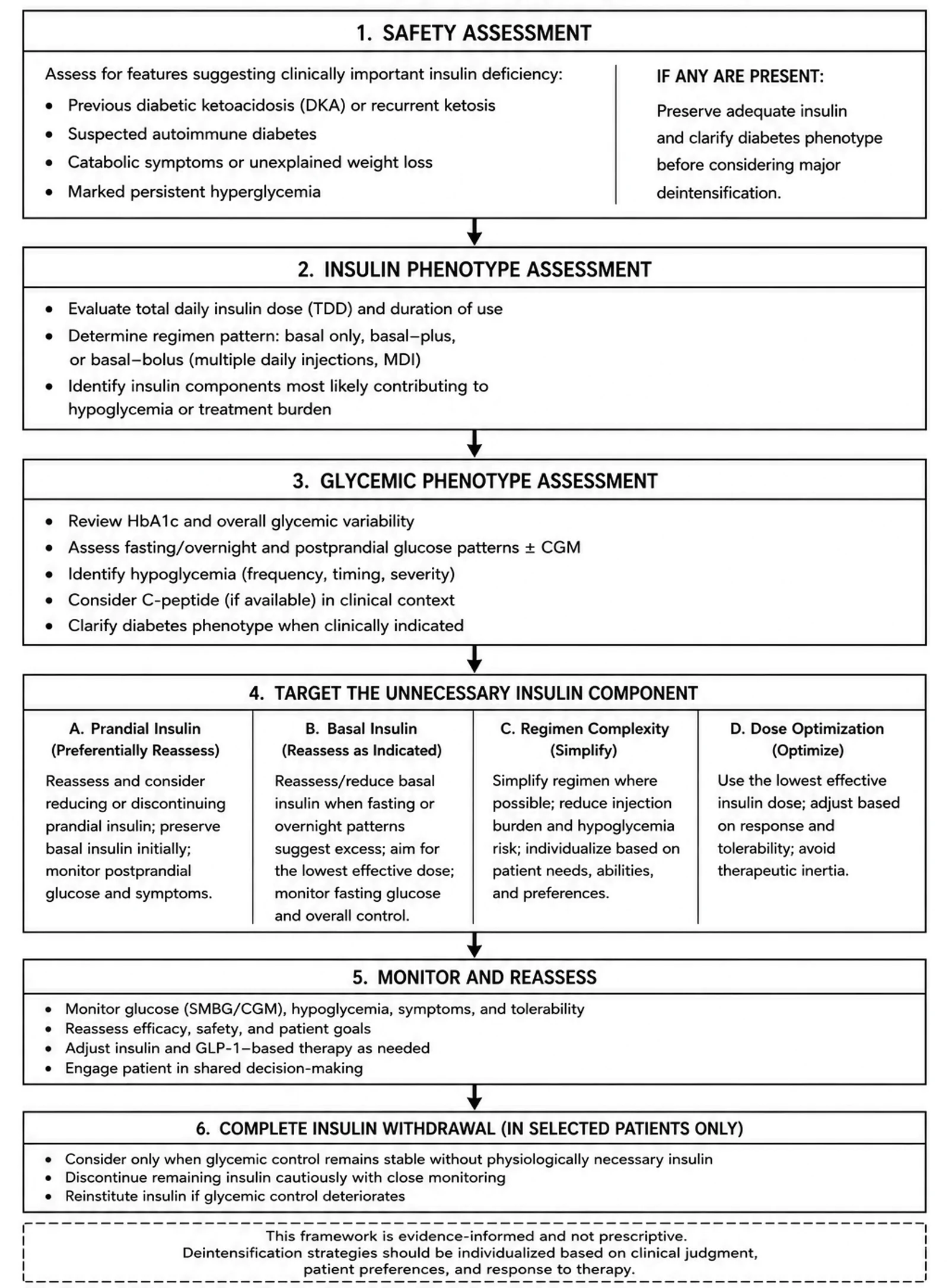
Evidence-informed framework for insulin deintensification after GLP-1-based therapy in type 2 diabetes This is an original author-created schematic and is not reproduced or adapted from another published source. The sequence is intended to support iterative clinical reassessment and does not represent a prospectively tested mandatory sequence of treatment steps. The framework is an authors' synthesis and has not been prospectively validated as a treatment algorithm. Abbreviations: CGM, continuous glucose monitoring; DKA, diabetic ketoacidosis; HbA1c, glycated hemoglobin; MDI, multiple daily injections; SMBG, self-monitoring of blood glucose; TDD, total daily dose; GLP-1, glucagon-like peptide-1.

How much insulin should be reduced?

Published deprescribing recommendations and insulin-adjustment protocols provide empiric starting strategies rather than validated universal dosing rules. For patients receiving basal insulin alone, an initial reduction of approximately 20% has been proposed when baseline HbA1c is ≤8%, provided the overall glucose profile supports dose reduction. In intensive regimens, clinical-study protocols have used reductions of up to approximately 25% of basal insulin and 50% of bolus insulin [16]. These values should not supersede glucose patterns, hypoglycemia risk, insulin requirements, or evidence of clinically important insulin deficiency.

These percentages should not be interpreted as automatic reductions whenever GLP-1-based therapy is initiated. Most GLP-1-based therapies are started at a lower dose and titrated progressively, so their full glucose-lowering effect may not be present at treatment initiation. The magnitude and timing of insulin reduction should therefore reflect baseline glycemia, hypoglycemia risk, the insulin component being targeted, current insulin requirements, the specific GLP-1-based therapy and stage of dose titration, and available glucose-monitoring data. Further reductions should follow demonstrated glycemic response rather than anticipated future efficacy [16].

Monitoring and reassessment

Deintensification should be treated as a monitored therapeutic trial rather than an irreversible decision. The practical sequence is reduce → monitor → confirm response → further reduce, maintain, or restore insulin. CGM can reveal overnight, fasting, postprandial, and hypoglycemic patterns that HbA1c alone cannot identify; structured self-monitoring of blood glucose (SMBG) remains a reasonable alternative [18]. Conventional CGM metrics have not been prospectively validated as thresholds for insulin withdrawal.

When deintensification does not succeed

Insulin reduction should be paused or reversed when glucose patterns show persistent clinically important deterioration rather than a transient isolated elevation. Particular concern should arise with symptomatic or sustained hyperglycemia, development of polyuria or polydipsia, unexplained weight loss or other catabolic features, ketonemia or ketonuria, or metabolic deterioration suggesting clinically important insulin deficiency. Ketosis or diabetic ketoacidosis requires prompt insulin-based management [1,15]. In less acute circumstances, restoration or escalation of insulin should be guided by the persistence and pattern of fasting and postprandial hyperglycemia, individualized glycemic targets, and the stage of GLP-1-based therapy titration. Available evidence does not establish a single glucose concentration or fixed duration of hyperglycemia that universally mandates insulin reintroduction.

Not every patient will be able to simplify insulin therapy to the same extent. Potential reasons include advanced beta-cell dysfunction, higher residual insulin requirement, progressive diabetes, inability to tolerate or maintain effective GLP-1-based therapy, intercurrent illness, glucocorticoid exposure, surgery, or metabolic stress. Insulin may need to be restored after an attempted reduction. Reintensification represents appropriate physiological reassessment when glucose control demonstrates that insulin remains necessary.

Limitations of the current evidence

Study populations differ substantially in baseline glycemic control, diabetes duration, insulin requirements, insulin regimen, GLP-1-based therapy, and eligibility criteria. Definitions of deintensification are inconsistent, and follow-up durations vary. Several direct withdrawal studies are relatively small and involve selected populations; TRANSITION-T2D, for example, enrolled 60 participants with HbA1c ≤7.5% and total daily insulin ≤120 units/day [9]. Observational studies remain susceptible to treatment-selection bias, confounding by indication, differential follow-up, and clinician-dependent decisions. Findings from one GLP-1-based agent should not automatically be generalized to another. No prospectively validated model currently identifies which patients can safely discontinue prandial or all insulin. As a narrative review, this article did not employ formal risk-of-bias tools or GRADE methodology, and the proposed evidence hierarchy therefore represents qualitative clinical interpretation rather than a formal certainty-of-evidence assessment. The proposed clinical framework integrates heterogeneous sources of evidence and has not itself been prospectively validated.

Evidence gaps and future directions

Immediate versus staged prandial withdrawal requires direct randomized comparison. Prospective prediction models are needed to determine whether insulin requirement, diabetes duration, HbA1c, C-peptide, and CGM phenotype can reliably identify successful candidates. The incremental value of C-peptide requires clarification. CGM-guided adaptive deintensification algorithms should be prospectively tested. Long-term trials should distinguish prandial withdrawal from complete insulin withdrawal and measure insulin reintroduction, hypoglycemia, treatment burden, quality of life, and durability. Comparative studies should determine whether the greater glycemic and weight effects of dual GIP/GLP-1 receptor agonism translate into greater, faster, or more durable insulin deintensification than selective GLP-1 receptor agonism. Prospective studies should also evaluate whether phenotype-based, iterative frameworks such as the one proposed here improve safety, treatment burden, and durability compared with usual clinician-directed insulin adjustment.

## Conclusions

Glucagon-like peptide-1 (GLP-1)-based therapies have changed insulin management in type 2 diabetes by creating opportunities not only to avoid further insulin intensification but also to simplify established complex regimens. The strongest direct evidence supports reduction or withdrawal of prandial insulin in appropriately selected patients, whereas evidence supporting complete insulin withdrawal remains less extensive.

Basal and prandial insulin should therefore be reassessed separately. No single HbA1c value, insulin dose, or C-peptide threshold can determine whether deintensification will succeed. A practical approach should instead integrate glycemic phenotype, current insulin regimen, hypoglycemia risk, probable endogenous insulin reserve, and the patient's evolving response to GLP-1-based therapy. CGM or structured glucose monitoring can facilitate sequential adjustment.

Insulin deintensification should be considered a monitored, individualized, and reversible therapeutic process. Further reduction, maintenance of a simplified regimen, and reintensification are all legitimate outcomes when supported by the patient's glucose physiology. The objective is not insulin avoidance, but the least complex insulin regimen necessary to maintain safe, individualized glycemic control.
